# Spatial Distribution of COVID-19 and Comorbidities in New England

**DOI:** 10.1093/geroni/igab046.2720

**Published:** 2021-12-17

**Authors:** Chae Man Lee, Taylor Jansen, Shu Xu, Maki Karakida, Frank Porell, Nina Silverstein, Elizabeth Dugan

**Affiliations:** 1 UMass Boston, Boston, Massachusetts, United States; 2 University of Masschusetts Boston, Dorchester, Massachusetts, United States; 3 University of Massachusetts Boston, University of Massachusetts Boston, Massachusetts, United States; 4 University of Massachusetts Boston, Boston, Massachusetts, United States

## Abstract

At the onset of the COVID-19 pandemic, the Centers for Disease Control and Prevention (CDC) identified chronic conditions which elevated the risk of COVID-19 complications: chronic obstructive pulmonary disease (COPD), diabetes, heart disease, kidney disease, and obesity. The aim of this study is to visualize the spatial distribution of confirmed cases of COVID-19 and local rates of comorbidities in CT, MA, NH, and RI and to identify the spatial clustering of hot spot between COVID-19 cases and rates of a summary measure of comorbidities across communities. This study collected data from state’s departments of public health in 4 New England states of confirmed COVID-19 cases as of February 25th, 2021 and extracted community-level rates of comorbidities among adults age 65+ from recent Healthy Aging Data Report (www.healthyagingdatareports.org). Results showed that the cities Bridgeport, CT (n=14,637), Boston, MA (n=57,912), Manchester, NH (n=9,658), and Providence, RI (n=26,792) had the highest rates of COVID-19 and the highest population density. The GIS based map illustrated that the largest cities with the highest population densities had both relatively high incidences of COVID-19 and heavy burdens of comorbidities. This study found that the hot spot areas of COVID-19 were observed in communities with the highest chronic disease burdens. These hot spots of COVID-19 and comorbidities are areas where resources (testing, masks, vaccines) should be surged to protect the community. The identification of hot spots may motivate residents to take every mitigation step to prevent and control COVID-19.

